# Some Rambling Thoughts on Medical Europe

**Published:** 1914-01

**Authors:** Dunbar Roy

**Affiliations:** Atlanta, Ga.


### SOME RAMBLING THOUGHTS ON MEDICAL EUROPE.

By Dunbar Roy, A. B., M. D., Atlanta, Ga.

Few American physicians and surgeons appreciate fully a trip to medical Europe. Not that they cannot obtain good medical work in this country, but it broadens one's experience to see how things are done by other nationalites where such work is frequently so systematized that it is readily accessible to the medical man. This is certainly true of medical work in Vienna. For post-graduate work in any medical line I know of no place which offers the advantages and opportunities which is afforded in this Austrian city. For years it has been the Mecca for American medical men, so much so that at present courses are given entirely in the English language and controlled entirely by the American Medical Association of Vienna. It is not exaggeration to say that there are constantly in Vienna, doing medical work an average of two or three hundred American physicians and surgeons. It was my good fortune last summer to spend a month in this city doing some special work and at the request of its Editor I have gotten down a few observations on Medical Europe which may be of some interest to the readers of the Journal-Record of Medicine.

When the medical man arrives in Vienna he would do well to immediately locate himself at the rooms of the American Medical Association which is close by the old general hospital and which is not only the center for obtaining knowledge of the various medical work which he desires, but it also a social club where one meets not infrequently old acquaintances or at least men who are worthy medical confreres. In one of the rooms of this association you will find posted all the various medical and surgical courses which are available and in this way one is able at the very beginning to probably find the work he wishes. You may not always be able to obtain a place in such courses, but it gives you a knowledge of how to obtain the same after you have been in Vienna a few days. These courses are usually limited to a certain number of men, but one soon finds out that much of the special work he desires can be obtained privately if he will only pay for it.

It is not my intention to give an account of this work which is being done in Vienna by the American Medical Association, sufficient is it for me to say that the association publishes a yearly book which gives all of this information and which can be obtained by writing to it in Vienna.

Nowhere in the world can the Otologist obtain such valuable work in diseases of the internal ear as can be obtained in this city. The names of Barany, Alexander Neumann, Ruttin, Bondy and others are intimately associated with this work and when we realize that we can obtain such work directly from these men, we must feel the value of a trip to this city.

Operative courses on the cadaver can be obtained with great satisfaction and he who would desire to perfect himself in the technique of any operation or even do original operative work must realize the value of this opportunity. The old general hospital in Vienna is still in daily use, but gradually they are building a more modern hospital at a point not far removed but a much better location. Some of the Clinics already removed to this new location includes the Medical Department of Von Noorden which is perhaps one of the most complete in all Europe. Unfortunately Prof. Von Noorden has resigned from this clinic and will remove to Frankfurt, due it is said to his inability to obtain sufficient appropriations to carry on the work in a manner satisfactory to himself. This clinic certainly offers wonderful opportunities to the medical man who has some knowledge of the great work done by Von Noorden on dietetics and metabolism.

Another clinic removed to this newer location is that of the Childrens under the direction of Von Pirquet. This building or hospital for children's diseases is one of the best to be found anywhere and has every modern improvement. For instance, the wards containing the infectious cases are so arranged with glass that the children can be examined without the physician coming directly in contact with the cases every time the rounds are made. The name of Von Pirquet assures one of the original work which is done in such an institution.

The Nose and Throat clinic has also been removed to this new location and is now housed in a most modern up-to-date building. Prof. Chairi, who is at the head of this department, has a remarkably large clinic of interesting cases. The clinic room where the examination of patients is made by the students of the University and post-graduate workers, is one of the most complete and satisfactory which we have ever seen. I wish time permitted me to give a detailed account of this arrangement for it is eminently satisfactory for the work intended.

In the old hospital there still remains many of the pioneer clinics. The surgical department made famous by the name of Billroth still offers great clinical work to the post-graduate man.

Perhaps there is no larger or more varied clinic than the skin clinic of Prof. Finger. This clinic has long been held in high repute by Dermatologists ever since the days of Hebra and Kaposi and even to-day it is exceedingly popular with physicians of all nationalities.

The first University Eye Clinic under the direction of Prof. Fuchs still remains one of the best in Europe. The material is very large and the very name of Fuchs would give it the stamp of scientific thoroughness. His First Assistant and Chief of Clinic, Dr. Josef Meller, is probably one of the best teachers to be found anywhere and his operative course on the eye is one of the most popular courses given in Vienna.

Prof. Dimmer of the second University Eye Clinic is also a man of some note while his First Assistant, Dr. Lauber, gives the best ophthalmoscopic course that can be found in all the world. He is exceedingly popular with Americans due greatly to his complete mastery of the English language. It will be unfortunate for American physicians to know that Dr. Lauber resigned on October 1st from this Clinic and in the future will be only a Privat-docent. Some slight feeling of jealousy is probably the cause and I understand that much of this same feeling is quite prevalent in the medical department of the University.

The Otological Clinic made famous by the names of Politzer and Gruber is still one of the best in all Europe. Politzer still takes an active interest in otology although his active work with the University ceased several years ago. Prof. Unbantschitz is the head of this department and with the aid of Bondy and Buttin, he has certainly made it the Mecca for the study of Otology by physicians all over the world.

Dr. Buttin is perhaps the most popular teacher among the American physicians in Vienna and it is frequently quite difficult to obtain work under this brilliant man. He is a natural-born teacher and has the greatest faculty for imparting this knowledge to others.

The great advantage to be found in regard to medical work in Vienna is that the same is systematized and accessible. You do not have to travel from one portion of the city to another in order to obtain different kinds of work. No time is lost and all of the clinics are in close radius of each other.

Not so in London, for here we have a city of immense distances where you go from one hospital to another with great difficulty and with much loss of time. If the immense clinics in all lines of work in London could ever be unified they would certainly offer the best advantages in the world to the medical man, but unfortunately London is so overwhelmingly large that I do not believe that such a realization could ever be imagined. Paris also offers the American physicians most excellent clinics, but like London they are scattered.

I had the pleasure of visiting the Skin Clinic at the Saint Louis Hospital. This clinic is probably the best of its kind in the world and it seems strange that the French people should be so offlicted with such rare skin diseases. While not a Dermatologist, I would certainly advise all those working in this line to try and make an occasional visit to this large and wonderfully varied clinic. Unless you speak French, however, you will be at a great disadvantage as this was the only place I found where the medical men in attendance did not also speak English.

Berlin offers great inducements also to the medical man. Unlike Vienna, the clinics are scattered and not nearly so accessible. The amount of work done at the Charity Hospital is immense and offers great clinical advantages to the post-graduate worker. I cannot fail to mention the new Rudolph-Virchow Hospital and its immense grounds. Without doubt this is certainly the realization of the ideal in hospital construction and management. It is situated some distance from the real center of Berlin but one is wonderfully repaid after he has reached the hospital. Every department of medicine and surgery is represented and he who could spend some time in this hospital would certainly have rare opportunities for medical work.

In concluding, these random thoughts I must mention a delightful visit which I paid to that quaint old Holland city of Utrecht. It has been one of my dreams ever since I began the study of Ophthamology, to visit the ophthamic hospital and clinic in this old Dutch city made famous by being the home and the field of labors of those two men distinguished in physiologic optics, Donders and Snelling. The former, whose work on Refraction will ever remain the standard book, and the latter, who continued the same good work and whose test-types are still accepted guides for every refractionist. Utrecht still posssses much of the quaintness and antiqueness of the Dutch civilization with its various canals running through it just as is seen throughout Holland. It is situated on the main line running from Cologne to Amsterdam and only one hour's ride from this latter city. I was there on a Thursday which is the great marriage day in Holland and we must have seen six marriage process-ions at the mid-day hour.

I had no difficulty in finding the Eye Hospital, for every-one knew the celebrated Prof. Snelling. The hospital itself was a three-story brick building, very neat, both without and within and most conveniently arranged for the purposes for which it was built. It is strictly an eye hospital and no other class of coses are treated. I was very cordially received by Prof. Snelling, Jr., who has now succeeded his father as the head of the hospital and as Professor in the University. The city of Utrecht makes considerable contribution for the main-tenance of this institution, but much is acquired by private contributions. The lower floor is devoted entirely to the out-door clinic which is conducted every morning from nine o'clock on by Prof. Snelling and his Assistants. One is impressed by the thoroughly painstaking work which is here done without noise or confusion. About ten-thirty he goes up to the operat-ing room and takes core of such cases as have been prepared for operation. He speaks good English and is quite courteous to all foreigners. I had the pleasure that morning of seeing him do two cateract extractions and an optical iridectomy. The operating room is simple in construction and the operating table nothing more than a stretcher resting upon woolen supports. Statistics show that the work done in this hospital compares fa-vorably with that done in hospitals of more elaborate structure. I afterwards made the rounds of the wards with Prof. Snelling and saw operative results well worthy of emulation. This visit was most interesting and the large marble bust of Don-ders in the front vestibule of the hospital makes one realize that the greatness of a man's work does not depend upon the great-ness of the city in which he lives.

